# Context specific realities and experiences of nurses and midwives in basic emergency obstetric and newborn care services in two district hospitals in Rwanda: a qualitative study

**DOI:** 10.1186/s12912-021-00793-y

**Published:** 2022-01-04

**Authors:** Aurore Nishimwe, Daphney Nozizwe Conco, Marc Nyssen, Latifat Ibisomi

**Affiliations:** 1 School of Public Health, Faculty of Health Sciences, University of the Witwatersrand, 1 Smuts Avenue, Braamfontein, Johannesburg, 2000 South Africa; 2 School of Public Health / College of Medicine and Health Sciences, University of Rwanda, P.O. Box 3286, Kigali, Rwanda; 3 Department of Biomedical Statistics and Informatics, Vrije Universiteit, Brussels, Belgium; 4 Nigerian Institute of Medical Research, 6 Edmund Cres, Yaba, Lagos, Nigeria

**Keywords:** Basic emergency obstetric and newborn care, Post-partum haemorrhage, Neonatal resuscitation, Nurse, Midwife, Rwanda

## Abstract

**Background:**

In low and middle-income countries, nurses and midwives are the frontline healthcare workers in obstetric care. Insights into experiences of these healthcare workers in managing obstetric emergencies are critical for improving the quality of care. This article presents such insights, from the nurses and midwives working in Rwandan district hospitals, who reflected on their experiences of managing the most common birth-related complications; postpartum hemorrhage (PPH) and newborn asphyxia. Rwanda has made remarkable progress in obstetric care. However, challenges remain in the provision of high-quality basic emergency obstetric and newborn care (BEmONC). This study is a qualitative part of a broader research project about implementation of an mLearning and mHealth decision support tool in BEmONC services in Rwanda.

**Methods:**

In this exploratory qualitative aspect of the research, four focus group discussions (FGDs) with 26 nurses and midwives from two district hospitals in Rwanda were conducted. Each FGD was made up of two parts. The first part focused on the participants’ reflections on the research results (from the previous study), while the second part explored their experiences of delivering obstetric care services. The research results included: survey results reflecting their knowledge and skills of PPH management and of neonatal resuscitation (NR); and findings from a six-month record review of PPH management and NR outcomes, from the district hospitals under study. Data were analyzed using hybrid thematic analysis.

**Results:**

The analysis revealed three main themes: (1) reflections to the baseline research results, (2) self-reflection on the current practices, and (3) contextual factors influencing the delivery of BEmONC services. Nurses and midwives felt that the presented findings were a true reflection of the reality and offered diverse explanations for the results. The participants’ narratives of lived experiences of providing BEmONC services are also presented.

**Conclusion:**

The insights of nurses and midwives regarding the management of birth-related complications revealed multi-faceted factors that influence the quality of their obstetric care. Even though the study was focused on PPH management and NR, the resulting recommendations to improve quality of care could benefit the broader field of maternal and child health, particularly in low and middle-income countries.

**Supplementary Information:**

The online version contains supplementary material available at 10.1186/s12912-021-00793-y.

## Background

Globally, an estimated 295,000 maternal deaths (211/100,000 live births), 2 million stillbirths and 2.5 million early newborn deaths occurred in 2017 [1, 2]. More than 90% of these deaths happened in low and middle-income countries (LMICs), like Rwanda [3]. The risk of mortality is highest during labor, where 46% of maternal deaths and stillbirths and 36% of neonatal deaths occur [4]. Preventable causes such as pre-eclampsia, obstructed labor, infections, post-partum hemorrhage (PPH), hypertensive disorders and newborns failure to breathe are frequent [5, 6]. To help reduce preventable deaths during the childbirth critical period, the WHO, UNICEF, and UNFPA established a set of seven key obstetric services or “signal functions” referred to as Basic Emergency Obstetric and Newborn Care (BEmONC) [7]. These include the management of post-partum hemorrhage; the removal of a retained placenta; the management of maternal sepsis; the management of hypertensive disorders; the management of prolonged labor; the management of complications of abortion, and newborn resuscitation [8]. The BEmONC services are mostly provided by nurses and midwives in low-resource settings [9]. The WHO further, advocates for all births to be attended by a skilled birth attendant [10]. The BEmONC services were introduced in health facilities of Rwanda in 2004 with over 150 healthcare providers and community health workers trained in BEmONC from 2004 to 2009 [8].

The Rwandan health sector has remarkably evolved, since 2017, 91% of births take place in healthcare facilities and attended by healthcare professionals [11, 12]. The Rwandan public health care system has a pyramidal structure consisting of five levels: referral/ university teaching hospitals at the top followed by provincial hospitals, district hospitals, health centres and health posts [13]. An additional cadre of trained community health workers (CHWs) was introduced to link pregnant women in the community with the health facilities [14]. Normal births occur at health post or health center level. In the event of complications, the pregnant woman is transferred to the district hospital where advanced equipments and services are available. The obstetric care services are predominantly offered by nurses and midwives with the help of physicians in case of potentially fatal complications. The pre-service training of nurses and midwives in Rwanda varies widely with technical (A2); advanced diploma (A1); baccalaureate (A0) and graduate (masters) training available as pathways to entry to nursing and midwifery practice [15]. The work responsibilities vary depending on the level of education with nurses and midwives having A1 level and above, being responsible for managing normal deliveries and birth-related complications.

Efforts to reduce maternal mortality in Rwanda have included initiatives to encourage more women to deliver at health facilities with the free birth care advantage and health insurances affordability with 10% out of pocket payment of all received services [16]. Also, The public healthcare structure ensured that obstetric care services are delivered at different levels in line with the levels of expertise required and this contributed to a significant increase of births taking place in healthcare facilities [17–19]. However, maternal mortality and neonatal deaths remain high despite these initiatives. The maternal mortality ratio for the period 2014-2015 to 2019-2020 was 203/100,000 live births [20] and PPH at 22.7% was the leading direct cause [21]. The neonatal mortality ratio was 19 deaths/ 1000 live births [20] and newborn asphyxia and its complications was the leading cause, accounting for 38% of all neonatal deaths [22, 23]. This situation makes PPH and newborn asphyxia the priority challenges in Rwanda. This suggests that while the rate of heath facility deliveries has risen, the quality of care in health facilities might be suboptimal.

The current paper, was derived from a broader study about implementation of an mLearning and mHealth decision support tool (Safe Delivery Application) in basic emergency obstetric and newborn care services in Rwanda [24]. The aim of this aspect of the study was to explore the participating nurses and midwives’ experiences and perceptions of obstetric care with a special focus on PPH management and NR. The objective of this aspect of the study was two-fold; one part was to elucidate from the participants’ explanations of the research findings [25] from the quantitative part of the research. The second part was to explore context specific factors that influence their work as obstetric care givers in their respective hospitals.

## Methods

### Study setting

The study was conducted in two out of the 12 district hospitals in the Eastern and Kigali provinces in Rwanda [11]. The selected hospitals are: Nyamata district hospital from the rural eastern province and Masaka district hospital from the Kigali urban province. Both hospitals had been offering BEmONC services for more than 5 years and had a high number of deliveries per year, with more than 6000 deliveries per year in Nyamata hospital and 7200 deliveries per year in Masaka hospital [26].

### Study participants

For the broader study, we invited nurses and midwives working in the maternity departments of the selected hospitals to participate in the study. Participants were selected using the following criteria: 1) having a work experience of at least 6 months in obstetric care, 2) being employed full-time in the selected hospital, and 3) willing to participate in the study. A total of 54 nurses and midwives participated in the baseline survey. For this qualitative study, 17 midwives and nine nurses were purposively sampled. The purpose was to select from participants who had participated in the survey and we were interested in having participants in line with the diverse roles in the maternity departments. Four focus group discussions were planned and the intended size of each focus group was a minimum of four and a maximum of six participants. According to Kitzinger and Barbour (1999), groups of this size are recommended for studies investigating health care systems and organization aspects [27]. In the present study, the number of participants who could attend on a given date per hospital determined the size of each group. Two groups in Nyamata district hospital consisted of six nurses and midwives each. While two groups in Masaka district hospital consisted of six and eight nurses and midwives respectively.

### Study design

This exploratory qualitative study was embedded in a three phased pre – post intervention research, investigating the effect of the Safe Delivery mHealth Application on nurses’ and midwives’ clinical decision making in the management of the two top frequent births complications in Rwanda (newborn asphyxia with its complications and post-partum hemorrhage). The management of these two birth complications include neonatal resuscitation and PPH management [24]. The qualitative research sought to obtain an in depth insight into the nurses’ and midwives’ experiences about offering BEmONC services in the selected district hospitals. The study adopted an exploratory qualitative research design using moderated focus group discussions [28] to both explore and describe the nurses and midwives’ reflections on the baseline survey results and their lived experiences in managing PPH and NR. The design was chosen as the nurses and midwives, who were delivering BEmONC services in the selected district hospitals, could offer a narrative explaining the baseline quantitative results and description of the phenomenon as they experienced it.

### Data collection

The researcher moderated four FGDs, two in each study hospital, using a semi-structured guide. Each FGD session explored both the reflections of the participants to the baseline research results and their experiences of delivering BEmONC services. For the reflections, the researcher presented two sets of baseline findings on PPH management and NR - the survey results of nurses’ and midwives’ knowledge and skills; and the six-month record review on maternal and newborn outcomes (Apgar score and PPH progressions) following PPH management and NR. The researcher invited participants to reflect and discuss the baseline findings, probing for their reactions and explanations. The second part of the FGD explored nurses’ and midwives’ experiences in delivering obstetric care services with a special focus on PPH management and NR in their respective district hospitals.

FGDs were conducted in July 2019 at both hospitals. The duration of the interviews ranged from 45 to 60 min. The interview guide (Additional file 1) was developed in English, translated to Kinyarwanda, and translated back to English in a blinded manner to ensure accuracy and equivalence [29]. Interviews were conducted in the language of the participants’ preference, which was Kinyarwanda. The first author conducted the FGDs with assistance of an experienced research assistant who assisted in taking notes and supervising the tape recording. To ensure the quality and adequacy of the FGD guide, it was pretested with one midwife and one nurse working in a similar department but at a different district hospital. Following the test, minor adjustments were made to the FGD guide. The minor adjustments included expanding one probe for more clarity, the probe was “peer-support” expanded to “possible peer-support in the work environment”. Questions were open-ended to allow participants to expand upon topics they felt were important. Interviews were held in private rooms that adequately accommodate 12 people at both Masaka and Nyamata district hospitals to ensure anonymity and confidentiality.

### Data analysis

Audio recordings of the FGDs were transcribed immediately following the discussions and then, translated from Kinyarwanda to English by a professional translator. Transcriptions were done following the True Verbatim method to accurately capture meanings, perceptions, and context [30]. Field notes were incorporated in the written transcripts as additional data. To ensure data quality, two researchers independently double-checked all transcriptions and translations. Data were analyzed using thematic analysis with a hybrid approach (inductive and deductive) [30], consisting of six steps: 1) familiarization with data; 2) identifying initial codes; 3) generating themes; 4) reviewing and defining themes; 5) coding the data; 6) organizing themes and write up. The thematic analysis is broadly used in qualitative research and aims to present the main elements of the participants’ descriptions [31]. The first author (AN) and one co-author (DNC) read all transcripts and developed the preliminary coding scheme together. Two interviews were double-coded by the first author (AN) and the co-author (DNC). Any inconsistencies were discussed and resolved to develop the final coding framework. The first author coded all remaining interviews transcripts. New themes, which could not be placed within the established coding framework, were also included in the analysis [32, 33]. Nvivo 11 Plus software was used in coding. The consolidated criteria for reporting qualitative research [34] guided reporting for this study (Additional file 2).

## Results

### Demographic information of the participants

A total of 26 nurses and midwives participated in the focus group discussions, 16 of whom were females and 10 males. More than a half were from Masaka district hospital (*n* = 14). The average age of participants was 32 years, ranging from 23 to 61 years. The level of education was predominantly the advanced diploma (A1) in midwifery with 14 of 26 participants having A1 level in midwifery. The majority of the participants had less than 10 years of experience in obstetric care (*n* = 18), and spent more than 10 h per week providing obstetric care (*n* = 20). Table 1 shows the demographic characteristics of the participants.

**Table 1:** Participants characteristics (*N* = 26)

	n (%)
**Hospital Affiliation**	
Masaka District Hospital	14 (54)
Nyamata District Hospital	12 (46)
**Gender**	
Male	10 (38)
Female	16 (62)
**Education Level**	
Midwife A0	3 (11)
Midwife A1	14 (54)
Nurse A0	2 (8)
Nurse A1	5 (19)
Nurse A2	2 (8)
**Years of experience in obstetrical care**	
1–5	9 (35)
6–10	9 (35)
> 10	8 (30)
**Weekly workload in obstetrical care (hours)**	
0–5	2 (8)
6–10	4 (15)
> 10	20 (77)
**Age, years, Mean (Range)**	32 (23 - 61)

*Abbreviations*: *%* Percentage

**Fig. 1:**
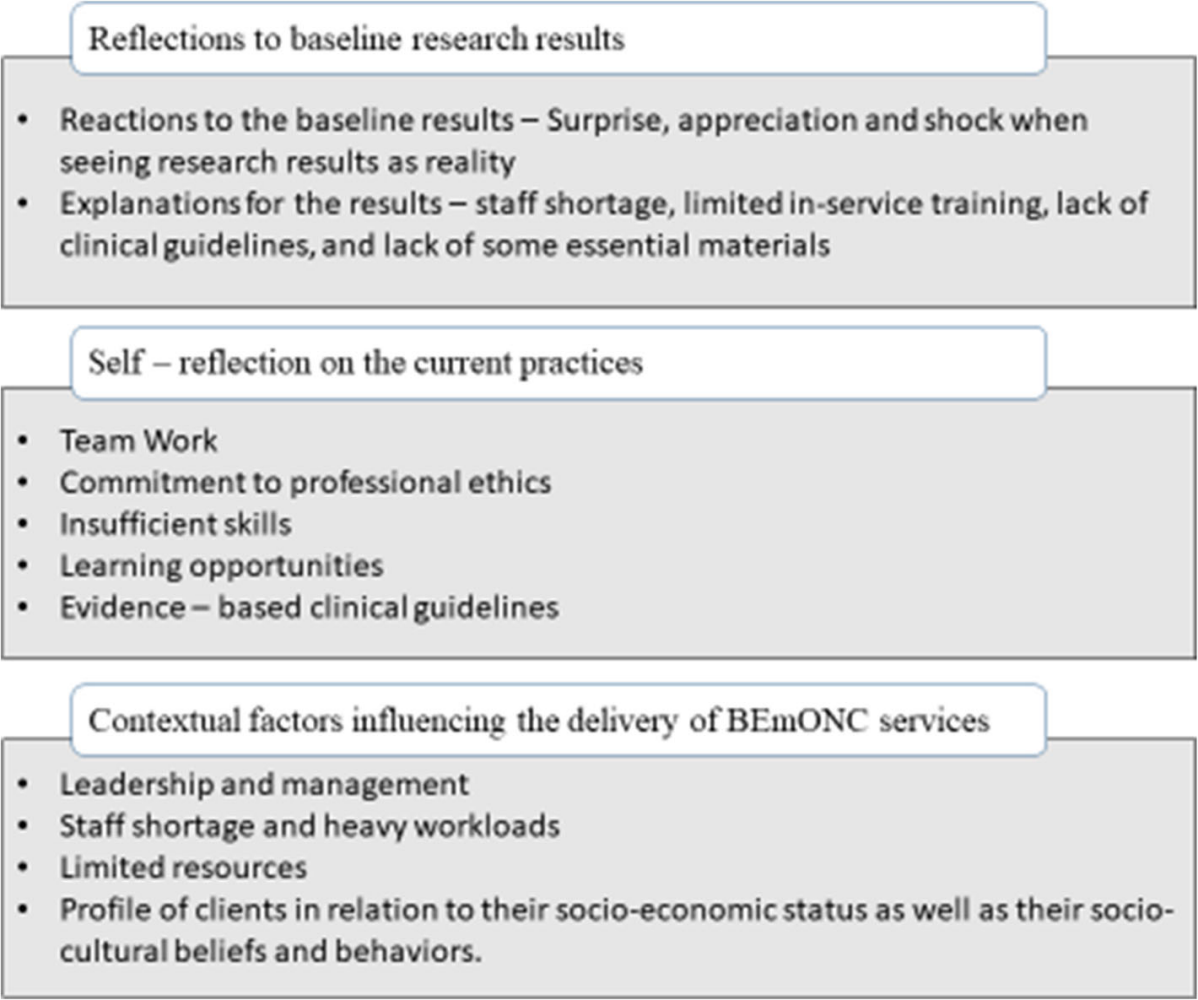
Thematic framework

The analysis revealed three main themes: (1) reflections to the baseline research results, (2) self-reflection on the current practices, and (3) contextual factors influencing the delivery of BEmONC services. During the discussion of results, verbatim quotations were used to support the themes and provide evidence. More details on the main themes and sub-themes (Fig. 1) are presented in the text below, and are illustrated by quotations from the four FGDs.

### Thematic framework

For each main theme, there were some associated key concepts that served as the sub - themes and resulted in the formation of the thematic framework shown in the Fig. 1.

### Reflections to baseline research results

This was a reflection about the baseline survey findings on the knowledge and skills in the management of PPH and NR, and on a 6 months’ record review of the BEmONC outcomes - Apgar score and PPH progressions. The survey results indicated that the average knowledge score was 47.2% and the average skills score was 59.9% among 33 midwives and 21 nurses interviewed for the survey [25]. The record review findings revealed an unstable newborn outcome (Apgar score < 7) following 10 min NR recorded in 62% (*n* = 78) newborn cases and an unstable maternal outcome (persistent bleeding ≥500mls) following PPH management recorded in 19% (*n* = 13) maternal cases. Under reflections to baseline research results, the sub-themes included reactions such as surprise, appreciation and shock, and explanations for the results which consisted of staff shortage, limited in-service training, lack of clinical guidelines, and lack of some essential materials.

Nurses and midwives admitted to being surprised by the survey and record review results. Some participants voiced appreciation of the research process, specifically receiving feedback on the baseline research results. They indicated that the research results presented to them, helped them reflect on their usual practice and to consider ways of improving the services that they provide.

*“It is the first time we get access to the findings from all the surveys that have been conducted here by the research people, this is good even though our performance was not good, I think we need to improve on our knowledge, get refresher courses or read a lot”***Midwife, Female-FGD1.**

Other participants said they were shocked by the realities of the performance of their departments, while others stated that they have always known that their performance was not optimum. The presented baseline results made some participants to consider ways to improve the situation.

*“I know our department is not best performing due to some challenges we face here in district hospitals like the shortage of staff and lack of some essential materials, but these findings are not good at all. What can we do to address this issue? … I think the hospital administration need to take the lead to ensure safe environment of work here and help us get in-service trainings, it is really difficult to get them.”***Midwife, Male-FGD4.**

Some participants thought that the reasons behind the low knowledge and skills scores was due to limited concentration while filling the survey questionnaires as they were thinking of the huge workload and this may have made them lose concentration.

*“I think that the figures reflect the reality. But, I think the research was conducted while people were thinking of the work ahead. As a result of this, it is possible that some participants didn’t take time to answer the questions properly with enough concentration. They might simply have ticked the answers without thinking about them. That is my opinion”***Midwife, Male-FGD1.**

Other possible explanations of the low knowledge and skills scores and the high records of unstable maternal and newborn outcomes, included staff related factors such as lack of skills where they describe the lack of in-service trainings as the main contributor.

*“When we compare the knowledge and skills scores with the records, …. We can see that the figures from the records review also are too high* (62% unstable newborn outcomes and 19% unstable maternal outcomes)*. It is still a fact that there is a serious problem regarding lack of skills or experience”***Nurse, Female-FGD2.**

Another participant said:

*“I think that the main reason is lack of regular and continuous trainings for us, … Continuous professional development is always needed not only to refresh but also to upgrade what one learnt at school, in other words, staff members regularly need training to get updates”***Midwife, Female-FGD3.**

Another reason highlighted was the shortage of staff in the maternity department. According to nurses and midwives, the health system plays an important role in the provision of care which later affects patients’ outcomes. The unstable maternal and newborn outcomes were attributed to the health system structure with the main highlight on the insufficiency of staff.

*“The biggest challenge is insufficiency of staff. Sometimes, you may need some assistance when dealing with newborn resuscitation or PPH and you fail to get someone to assist. In this case, you need to do everything by yourself and as a result you may not succeed in everything as it could have been if there were two of you”***Nurse, Male-FGD2.**

Other participants voiced the need for adequate clinical guidelines that would be accessible and visible in the time of need, especially in case of emergencies, to help in decision making when dealing with birth complications such as PPH and NR.

*“We need clinical guidelines to refer to regularly to provide better care to patients. The existing guidelines are not enough and not displayed in every labor room, … I think that the hospital should get more to have them displayed anywhere they may be needed”***Nurse, Female-FGD3.**

The lack of essential materials was mentioned as another reason why there were high records of unstable maternal and newborn outcomes.

*We might be in a situation where some materials, equipment and medicines are not available. Sometimes, there is a possibility of providing good service but when you fail to get what you need (materials) to provide such a service on time, there is a problem and you can’t succeed in your duties”***Nurse, Female-FGD4.**

### Self – reflection on the current practices

The participants’ narratives of lived experiences of providing obstetric care services in the study district hospitals revealed some insights into their current practices towards providing optimal BEmONC services. The discussions focused on team work, commitment to professional ethics, insufficient skills, learning opportunities and evidence-based clinical guidelines. Firstly, participants discussed the importance of peer-support for improved BEmONC services provision, particularly with regard to the management of PPH and NR. They acknowledged the presence of a team work spirit among nurses and midwives.

*“I am happy with the teamwork spirit in this hospital and I find this as motivation and support here. Though we have different tasks to perform here, I appreciate the way we work together”***Nurse, Female-FGD3.**

Likewise, nurses and midwives indicated that sharing responsibilities is a significant enabler of good service provision. They link it to the issue of shortage of staff and high delivery loads. The mechanisms of supporting each other despite everyone’s allocated daily responsibility make the work possible with the support of good and timely communication.

*“I like the fact that people working in maternity ward are good at communicating. I am saying this because when several deliveries are to take place at the same time, they would always call upon the coordinator to send in more staff members for support.”***Midwife, Female-FGD1.**

In addition, participants explained that their motivation which lead to their commitment to the professional ethics is a facilitator to better service provision. They discussed the value of wanting to save lives which keep them motivated to do whatever they can to care for women and newborns.

*“I would like to say that we are motivated because we always wish to save lives. Of course, nobody would wish to experience newborn asphyxia. It is bad for any staff member. You would even get blamed for that. We are motivated and this has good impact on what we do. You can see that people like their job very much. You will see that we come not because we have to come but to save lives of mothers and newborns and to ensure that the work is properly done”***Nurse, Female-FGD2.**

However, participants acknowledged the presence of insufficient skills to deal with birth complications among some nurses and midwives coupled with the lack of up to date clinical guidelines, which pose barriers to patient care. This was expressed as follows:

*“The challenge I would raise is limited skills. Maybe it is not an issue for everyone but it is there. Among five team members, you may only find two with the skills that are required for newborn resuscitation”***Midwife, Female-FGD4.**

Another participant stated that:

*“Some nurses and midwives may not have enough skills to support the patients. For example, when it comes to cervical tear, they might fail to know how to suture the tear, thinking that it is only done by a doctor and remember that there is only one doctor assigned to maternity service”***Midwife, Female-FGD2.**

Furthermore, nurses and midwives described the factors in respect of their professional development as significant contributors to the quality of obstetric care. The lack of in-service professional development courses was mentioned as a crucial barrier to quality BEmONC service provision. Participants stated a need to get access to regular in-service trainings and continuous learning opportunities to improve on their knowledge and skills which would positively affect patient outcomes. There was also a recommendation to promote the culture of reading among nurses and midwives.

*“The big problem is that most of us do not have access to trainings and/or in -service professional development courses. Also, the culture of reading is not in us. We just keep doing things as we have always been doing.”***Midwife, Male-FGD3.**

The study participants also mentioned the few learning opportunities that are available in the district hospitals which currently contribute to their continuous learning. These leaning opportunities include; mentorship sessions and clinical staff meetings. Participants agreed that those mentorship sessions improved their knowledge, skills and confidence, particularly with regard to maternal and child health care, and are key facilitators of evidence-based care provision.

*“We nowadays have mentors from Rwanda Association of Midwives (RAM) and Ingobyi Project who give training to clinical staff. Trainings are provided to doctors, nurses and midwives working in maternity. Trainees meet and work with these mentors every month. They give training in obstetric care. Their support is essential and it has improved our ability in providing better services to mothers and newborns. Those from Ingobyi have given training to four staff members so far, two of these four cascade the training to staff in health centres. Those from RAM together with Rwanda Pediatric Association come here every month to train three people per month.”***Midwife, Male-FGD1.**

In addition, several participants felt that nurses and midwives who have not yet participated in the mentorship program should be included in training when possible. For this to happen, the training frequency might be increased to reach more staff. Other related suggestions included pairing already trained staff with non-trained staff during work shifts to improve both care provision and learning. Participants also suggested that the mentorship sessions could focus more on birth complications such as PPH management and NR.

*“I would also like to say that a big number of staff members in maternity department have not yet participated in the mentorship program. The number is still too low. If the days of mentorship could be increased, everyone will get a chance to be trained. We need that the sessions focus more on PPH and neonatal resuscitation. Remember, some staff members are fresh graduates who only have knowledge without experience. I think they need a lot of continuous professional development trainings. Also having them on day or night shifts with the more experienced staff who have benefited from the mentorship would make the situation better”***Midwife, Female-FGD4.**

Also, participants discussed the importance of clinical staff meetings that happen every morning to discuss obstetric care management. The meetings involve the presentation of cases of patients managed in the previous night as well as the hospitalized patients. The meetings also focus on the management of particular conditions in critical patients with senior staff advising junior staff. Nurses and midwives acknowledge these meetings as learning opportunities with real patient case presentations. Thus, helping them to improve the service provision. Participants also indicated that they have other educational meetings twice a week which are beneficial to their knowledge and skills refreshment.

*“I think it is a good thing that we have staff meetings at hospital level to regularly discuss birth complications and related problems*. *We do not wait for these structured mentorship initiatives organized by external people. For example, in maternity ward, we also have educational meetings that are done on Wednesdays and Thursdays in addition to regular staff meetings that we have every morning. In these meetings, we talk about cases we have had and we thereafter have presentations about anything we think is useful. We often talk of PPH, eclampsia and helping baby breath. This is where emphasis is mostly laid to ensure that everyone working in maternity have basic skills in this. We also do some practices using the mannequins”***Midwife, Female-FGD2.**

Further, nurses and midwives discussed the role of evidence-based clinical guidelines in continuous learning and in the provision of evidence-based obstetric care. Participants stated that they need clinical guidelines to support their service provision. However, they pointed out the insufficiency of clinical guidelines as an important barrier to quality service provision. They indicated that the few clinical guidelines available are not displayed wherever needed.

*“I would like to add that clinical guidelines are important for us. Clinical guidelines are there but not enough. I think there is a need to have them available in more places including emergency area where we also have PPH cases to deal with. Those working with ambulance should also have the guidelines because they are the ones to take care of the PPH patient while being transferred. They should also be displayed in maternity, emergency and out-patient and even in surgery room and this should be done in a sufficient quantity. I don’t think they are available in health centres too. You may visit a place and notice that they don’t have a PPH guideline while they may have had two or three PPH cases in a month.*” **Midwife, Female-FGD3.**

Other participants indicated that the available clinical guidelines are not updated. They stressed that the availability of enough and up to date clinical guidelines would help them in clinical decision making while dealing with birth complications such as PPH and NR.

*“A staff member may not be in position to always remember what to do when there is a PPH or neonatal resuscitation. However, clinical guidelines are still few. Only one is displayed in the delivery room, it has been there for long ago and it is not updated.”* **Midwife, Female-FGD2.**

In addition, participants indicated the challenges with paper based clinical guidelines which are the only ones available. Participants cited the length, the difficulty to update and the deterioration with time as the main challenges of paper based clinical guidelines. They also stressed that it is hard for them to find time to read the non-summarized paper based clinical guidelines.

*“There are few clinical guidelines: some are in file boxes and others are displayed on the walls…, it is hard to update these paper based-clinical guidelines and they could get deteriorated easily as time goes. The ones in file boxes are too long and it is unfortunate we do not read them- maybe reading is so difficult! People are too lazy to read or maybe don’t have time to read. You will hardly see someone reading the clinical guidelines in files boxes”* **Midwife, Male-FGD4.**

Finally, nurses and midwives, in consideration of the evolving digital age, proposed to have the summarized electronic clinical guidelines which could be easily accessible and regularly updated.

*“I have read on internet that in developed countries’ hospitals, there exist some form of electronic clinical guidelines that people consult on computers or smartphones. And, I think the easy way for us would be to have those electronic clinical guidelines that will be summarized, easily accessible for everyone and could be updated as science evolves.”* **Midwife, Female-FGD1.**

### Contextual factors influencing the delivery of BEmONC services

Nurses and midwives indicated a number of context specific factors that influence the quality of care in BEmONC, particularly in the management of PPH and NR. Under contextual factors, the sub-themes included the realities of working in the context of district hospitals including the leadership and management, the staff shortage and heavy workload, the limited resources, the profile of clients in relation to their socio-economic status as well as their socio-cultural beliefs and behaviors.

The participants highlighted the contribution of the leadership and management in ensuring the quality of obstetric care in the hospitals. Participants described hands on leadership, provided by departmental managers, ensuring that services were well organized and that staff were assigned to where they were most needed. The good organization of service within the maternity department was perceived as key to better service provision.

*“We actually have a kind of task distribution and daily organization of the work by the maternity matron. When there is an emergency, for example in delivery room, those people in charge of different units get in touch and they may get support from each other by sending some members to help. The number of members to support will depend on the size of work to be performed. Such movements often happen between delivery room and hospitalization unit depending on cases.”* **Midwife, Female-FGD2.**

The participants described the support provided by the hospital management as enabling the service provision for the management of PPH and NR. They felt that the managers of the hospitals give a particular attention to maternal and child care which translate into more resources and supplies to the maternity department. They verbalized that:

*“The maternity heads in collaboration with the hospital managers do all they can to have all we need in stock. This include also PPH and NR emergency kits and all other kits. We organize a morbidity day event in which we discuss birth complications and the Director General also attends the event. They take into consideration all our challenges in the provision of maternity care”* **Midwife, Male-FGD4.**

*“What I can add is that we get a lot of support from the Hospital Management. Top management is very supportive and most importantly, the nursing leader is a midwife too. They are so sensitive about mothers. We usually get all equipments and drugs we need, even though sometimes, we run out of stock”* **Midwife, Female-FGD1.**

However, participants described staffing shortages as a significant barrier to care provision. They explained it was common in district hospitals for two midwives or nurses to cover the waiting room, the emergency room, the labor room and the recovery room.

*“There is a problem of workload. People here have too much to do. You may find two staff members in the maternity and when you have to attend to six mothers at once, you understand that it can’t work. As soon as a delivery has taken place, you immediately go to another mother without considering subsequent stages as you should and then monitoring PPH becomes hard. As a result, sometimes there may be some complications and you may fail to handle them on time”* **Midwife, Female-FGD2.**

Some participants stated that high number of deliveries per midwife made it difficult to perform well for every patient in need. They cited the poor staff/patient ratio, as an important barrier to good service provision in terms of dealing with birth-related complications. They stressed that the number of staff members is too low compared to the number of patients to take care of.

“*I think there is a problem of staff/patient ratio. Monitoring also becomes very hard due to insufficient staff members. How can two midwives assist three deliveries at once? Who can meanwhile attend to those in the waiting room? Sometimes, you may end up finding the ones you left in waiting room suffering. The problem of insufficiency in staffing is crucial”* **Midwife, Male-FGD3.**

Then again, nurses and midwives stated that the low number of doctors assigned to the maternity department is a challenge to better service delivery. This is partly linked to the shortage of staffs in district hospitals.

*“I think that availability of medical doctors is also a requirement for these cases of PPH and NR, however they are still few, one allocated to Maternity service daily”* **Nurse, Female-FGD1.**

Other participants felt that they need more specialist doctors like pediatricians when performing neonatal resuscitation and later for the recovery of the newborn.

*“Another area to improve on is that we should also have a pediatrician to work with newborn resuscitation. The newborn resuscitation should not only be done by a midwife and a nurse. Sometimes, the newborn also needs antibiotics and a pediatrician would be in a better position to prescribe them. I think this is worth noting too”* **Nurse, Female-FGD4.**

Furthermore, participants highlighted the issue of staff rotations in different services and staff turnover as barriers to better service provision. They expressed concerns about staff rotations that is implemented by the health system in district hospitals of Rwanda. Staff, particularly nurses, are rotated to different clinical services such as maternity, pediatric, surgery and sometimes rotating to or from maternity. As a result, the rotating nurses, like new staff may not be familiar with dealing with birth complications and they may take time to get used to the routine work. Also, staff turnover was indicated as an important barrier in consideration of staff moving from one hospital to another hospital. When staff turnover takes place the origin hospital loses an experienced staff.

*“We know well that we sometimes have staff rotations and staff turnovers in hospital- for example fresh graduates who have no experience and moving back and forth in different services. These new staff members might proceed with a given management of the birth complications without following the standard clinical guidelines”***Nurse, Male-FGD3.**

Further, nurses and midwives discussed some of the resources challenge they face when dealing with obstetric emergencies. They cited lack of physical resources, including supplies and equipment, as a barrier to care provision. Resources such as oxygen cylinders, heating lamps for newborns, and suction bulbs were often unavailable or non-functional.

*“…. We only have one suction bulb here, imagine if we have more than one neonatal who fails to cry after birth, what can we do with one piece only? When this happens, we face a serious problem. For the time being, we only have three penguins and one lamp…. These materials are not sufficient”***Nurse, Female-FGD1.**

Another participant said:

*“Another challenge is the problem related to availability and accessibility of some medications and materials. If I think of the number of deliveries we have here, they should match the quantity of equipment and materials needed for that purpose. I don’t understand how you can assist in 15 deliveries with only one heating lamp at night…. Sometimes, you may even fail to get oxygen cylinders because they are not there or not in sufficient quantities….”***Midwife, Male-FGD4.**

Also, some participants described the persistent shortages of uterotonics, antibiotics and intravenous fluids. When medications were out of stock, patients or their family members were asked to purchase medications from outside pharmacies or, in emergency cases, nurses or midwives sometimes purchased medications for patients.

*“We normally use oxytocin and cytotec. When we don’t have these drugs in our hospital pharmacy, the patient attendants go out to buy them in private pharmacy and it might be a bit too late to provide good service by the time they get back to us with these drugs”***Nurse, Female-FGD1.**

Furthermore, nurses and midwives described the limited number of ambulances at the health centers level as an important barrier to better service provision. They highlighted that they receive some of the patients already in critical conditions due to travel delays related to the lack of transport/ambulances.

*“The real problem starts at health centres because they may wish to transfer a mother with a complication but they fail to get transport for her. Some health centres do not have ambulances. You can see how much time it would then take for an ambulance to leave here and go to pick up that mother at a health centre. That ambulance will reach there when this mother is already in critical conditions”***Midwife, Female-FGD2.**

Another contextual factor highlighted by nurses and midwives is the financial barrier among the patients. Participants described how financial barriers made service provision challenging for some patients. The lack of financial means for some mothers make them delay to present themselves to the hospital. And by the time they come, they are in a critical condition.

*“Some patients are coming to hospital too late due to financial problems and in this case, both the mother and her baby have started to have some complications. It is a fact that sometimes, you fail to save a newborn’s life not because you didn’t have enough skills but because you started attending to the mother when the situation had gone beyond boundaries”***Nurse, Male-FGD1.**

Participants also indicated that some patients cannot afford drugs and nurses and midwives cannot assist all of them with the emergency kits available which contain few emergency medications and materials. Other patients also get issues in transfer when they cannot afford the transfer fee.

*“Another thing is lack of financial means by some patients to afford drugs. It is a fact that we have emergency kits to assist such patients but you may not get everything in those kits that is required for all the cases”***Nurse, Female-FGD2.**

Another participant stated that:

*“Sometimes we may have patients who cannot afford the services we provide. For instance, when we have a serious case to transfer, such a patient may not afford the transfer fee…When then time is being wasted discussing this, a patient might get in worse conditions”***Midwife, Female-FGD4.**

On the other hand, nurses and midwives described low literacy and social-cultural and beliefs behaviors among mothers who visit the hospitals as contributing factors to the outcomes of delivery. Participants indicated that the use of traditional medications was prevalent in local communities. They indicated that most of the mothers coming to the hospitals for delivery, first take traditional medicines and these traditional medicines make the newborn suffer. As a result, the baby is born very weak and subject to neonatal resuscitation which is mainly hard to manage because the newborn in most cases has inhaled these traditional medicines.

*“Nowadays, based on my personal observation, some mothers come to hospital after taking some traditional medicines and this leads to aspiration syndrome and make the resuscitation of a newborn difficult”***Nurse, Female-FGD3***.*

Another participant said that:

*“I can add that there is a problem of literacy with the mothers we work with. They think that traditional healers help them to have live newborns. A large number of mothers who come here for delivery, they first take those traditional medicines. Such traditional drugs make the babies suffer a lot”***Midwife, Female-FGD3***.*

Other participants described an overall lack of awareness about the value of medical care during childbirth among some mothers who think that medical care is not enough that it has to be supplemented by traditional healers. There are misconceptions surrounding childbirth that mothers should take traditional medicines before delivery if they want to come up with a live newborn. Further, nurses and midwives expressed the need of health programs to increase the awareness of value of medical care during childbirth among the population.

*“I think there should be initiatives to tell the mothers to stop taking traditional medicines. They should let these mothers know about the negative effects of these medicines and ask them to stop such practices. This is a common practice with mothers who come here and it really has a negative effect on the newborns they give birth to”***Midwife, Female-FGD2.**

## Discussion

Our research contributes to existing literature insights from in-depth exploration of the perceptions and experiences of nurses and midwives on obstetric care services particularly, with the management of birth related complications such as PPH and NR. The participants reflected on survey results and offered diverse explanations of the results. Participants also shared their lived experiences of managing PPH and NR and gave their opinions on what they believe is influencing their work as obstetric care givers in their respective hospital. Our findings illustrate the complexity of the process of managing birth-related complications in district hospitals of Rwanda. The three main themes from the FGDs with nurses and midwives were: reflections to baseline research results, self-reflections on the current practices, and contextual factors influencing the delivery of BEmONC services.

### Reflections to baseline research results

The presented baseline research results were accepted as a true reflection of the reality of practice. Participants were shocked by the low knowledge and skills scores as well as the high number of unstable newborn and maternal outcomes. This made them reflect on their performance as a department and consequently provided possible explanations of the research results. Among the provided explanations, there were challenges such as staff shortages, limited in-service trainings, limited access to clinical guidelines, and lack of some essential materials. Similarly, a number of studies has explored the barriers to and facilitators of emergency obstetric care provision [35–38] and reported similar challenges in multiple health facilities in LMICs. Therefore, engaging nurses and midwives on how they perceive and reflect on obstetric care services reveals realities of practice that decision makers would consider when putting in place strategies to improve obstetric care particularly in low and middle income countries.

Moreover, other participants explained that their low scores in the knowledge and skills surveys were linked to their high workload. They indicate that their failure was due to the fact that they did not fully concentrate while filling the survey questionnaires because they were thinking of the huge work ahead. In the same vein, a study that explored perceptions of emergency obstetric care providers in Malawi on the critical factors of staff shortages and workload in their health facilities, reported the shortage of staff coupled with the unmanageable workloads that exceeded their capacity to cope as key contributors to the substandard provision of obstetric care [39].

### Self-reflections on the current practices

Our findings revealed that effective teamwork is a key facilitator to better provision of care. The participants acknowledged mutual support when managing PPH and NR and valued their attitudes of sharing responsibilities. Comparable findings have been seen in a study which focused on optimizing teamwork and leadership in reducing risk in maternity [40]. Another study which documented lessons from a series of research on teamwork, leadership and team training with regards to what makes maternity teams effective and safe [41] reported that teamwork is essential for managing complex pregnancies. Similarly, participants in the study perceived that peer support make a noticeable difference to team effectiveness and patient safety during obstetric emergencies [41]. Evidence of benefits of teamwork in maternal and newborn care have been indicated by several researchers [42–44]. Hence, teamwork is recommended when dealing with PPH and NR. Also, in the current study, the commitment to the professional ethics has been linked to teamwork and shown to promote staff motivation and improve obstetric care services.

Additionally, in the current study, on site mentorship sessions and clinical staff meetings, were identified as learning opportunities and key facilitators of evidence – based care provision. They helped nurses and midwives to update their knowledge and skills through critical discussions of specific cases and hands on practices with mentors. Other mentorship initiatives in partnership with the Human Resources for Health (HRH) Program have also been beneficial to the improvement of health service delivery in Rwanda [45, 46]. Similarly, a study in India found that a seven-month simulation-enhanced mentoring program for nurses and midwives from 320 primary health clinics improved their skills and confidence in managing common obstetric and neonatal complications [37]. Similar findings were documented in a prospective before-and-after study in Malawi, which evaluated the impact of a hospital-based mentoring program about EmONC on knowledge and skills among health providers [47]. The study has reported an increase of both short- and longer-term improvement in BEmONC knowledge and skills [47]. However, more research is required to assess whether the improvement in knowledge, skills and confidence leads to changes in practices that improve maternal and neonatal outcomes.

However, this study has identified barriers related to staff development. Study participants acknowledged having insufficient skills in PPH management and NR. They linked this challenge with the lack of professional development courses and the lack of culture of reading. Nurses and midwives emphasized on their need of continuous learning opportunities as the medical science evolves with new discoveries and approaches to deal with birth complications. They mainly highlighted how difficult it is to access continuous learning opportunities. Other studies have similarly noted that access to continuing professional development opportunities for nurses and midwives is often difficult, especially in LMICs [48, 49]. Filby et al. (2016) found that the lack of investment in continuing education prevents quality midwifery care [36]. Opportunities to address the in-service training challenges are available through short courses using competency-based learning curricula. However, context realities should be considered in terms of costs. The adoption of innovative methods of on job learning that utilize technology such as eLearning and mLearning could be adopted where face to face trainings are not accessible and less affordable.

In addition, the access to relevant and up-to-date clinical guidelines was another important point of discussion raised by nurses and midwives. Study participants acknowledged the importance of evidence-based clinical guidelines in continuous learning and its support in decision making at the point of care. However, the accessibility is still a challenge, as the available paper based clinical guidelines are few and not updated. Nurses and midwives also indicated that it is hard for them to find time to read the guidelines filed in cupboards. Likewise, a recent study mapped evidence on human resource-related challenges to quality facility-based newborn care provision by nurses and midwives [48] and reported the lack of access to current evidence-based guidelines as a common complaint in LMICs [48]. Other studies indicated that the care during labour and childbirth following evidence-informed clinical guidelines is associated with better patient outcomes [50–53]. Guidelines change regularly and thus dissemination and methods of updating nurses and midwives on new knowledge are essential. But, simple dissemination of paper-based clinical guidelines is ineffective as it was noted by our study participants. Electronic clinical guidelines and electronic decision support tools could be envisaged leveraging the rapidly increasing rates of smartphone access and mHealth technologies. Yet, additional research is needed on electronic clinical decision support tools, particularly in LMICs.

### Contextual factors influencing the delivery of BEmONC services

The current study shown that the good leadership and management of the district hospitals is an essential facilitator of care provision for PPH management and NR. Participants referred to the good leadership in consideration of service organization (distribution of tasks and allocation of duties). The hospital support was an additional facilitator of care provision, particularly with regards to achieving sustainable improvements and the particular support in terms of resources and suppliers. Katie et al. (2013) similarly suggested that management practices coupled with leadership behaviours can make a huge difference to outcome and experience for women and their companions [40]. Other studies have also documented the benefits of hospital leadership on patients’ outcomes [54, 55]. Therefore, leadership is considered a core element for a well-coordinated and integrated provision of care.

However, the staff shortage was highlighted as a significant barrier to good service provision for PPH management and NR. Nurses and midwives indicated that the number of staff was very low compared to the number of patients to take care of. Similar findings have been reported in other LMICs [39, 56]. A study by Dani et al. (2020) found that healthy term infants’ neonatal outcome is negatively associated with a low midwife-to-infant ratio [57]. Similarly, Christine et al. (2011) assessed the association between nursing staffing and patient outcomes and reported that inappropriate nursing staffing was linked to negative patient outcomes [58]. Other studies have also documented the effect of staff shortage on adverse events and increased medical costs [56, 59–61]. Moreover, Sosa et al. (2018) proposes the availability of a one midwife to one woman ratio for all women in labour [62]. Thus, an appropriate patient: staff ratio is revealed to be a determinant of quality obstetric and better maternal and newborn outcomes.

Also, the limited number of staff coupled with staff rotations and turnovers and a limited number of doctors posed significant barriers to PPH management and NR services in the two district hospitals under study. Similar human resource constraints have been reported in other LMICs [63]. Our study findings suggest that there is a problem of shortage of staff which leads to the fact that few nurses and midwives available work under pressure with less time spent with each woman leading sometimes to a lack of proper care. The resulting low quality of services is unpreventable. Therefore, one of the priorities is the need that policy makers put in place strategies that will help to increase the number of trained staff in obstetric care.

Other challenges identified in this study included the lack of some necessary medical supplies and problems related to the referral system such as few ambulances at the health center level. These findings were congruent with a report from a study conducted in India which revealed physical resource shortage as a barrier to the provision of optimal obstetric and neonatal emergency care [37]. Another study which identified barriers associated with the referral of emergency obstetric cases to a national referral centre in Ghana reported the problems with referral transportation system highlighting limited ambulances as a critical barrier to obstetric care service provision [64]. The quality of care of hospitals providing obstetric care has also been shown to be affected by a lack of required equipment and drugs as demonstrated by studies conducted in past [35]. Therefore, understanding these challenges from the healthcare providers’ perspectives is important and informative to policy makers in order to put in place context specific strategies to improve obstetric care.

Additionally, in this study, financial barriers were found to play a crucial role in delaying the access to medical care when needed. Nurses and midwives discussed on how patients with low economic status were likely to reach the hospital late and in critical condition. Other patients were not able to afford drugs and got issues in transfers. Geleto et al. (2018) in his systematic review about access and utilization of emergency obstetric care (EmOC) at health facilities in sub-Saharan Africa [65] reported that financial barriers prevent mothers to access EmOC when needed. Other studies have also noted the burden of financial barriers to maternal care particularly in Sub-Saharan Africa [63, 66]. Initiatives to reduce maternal mortality in Rwanda have included the facilitation of access to health care services with the help of the national medical insurance scheme [67]. The current hospital delivery rate in Rwanda is 91% [11]. However, some women still face financial barriers to afford other required fees like transfer fees from health centers to district hospitals which delay the care they receive for instance in case of PPH. There is need for more support to the very poor patients for healthcare services.

Moreover, the current study identified barriers related to patient challenges such as social-cultural beliefs and behaviors. With regards to cultural beliefs and behaviors, nurses and midwives were referring to the use of traditional medicines by women while these traditional drugs have a negative impact on the newborn health outcome. Similar findings were documented in a study conducted in Zambia which revealed multiple interconnecting factors linked with cultural beliefs that influence traditional medicine use among pregnant women [68]. Women believed that traditional medicines help them with labour induction, and prevention of childbirth complications [68]. From the modern medical profession perspective, these traditional medicines are always associated with adverse health outcomes, specifically for the newborn [68]. A lack of awareness about the value of medical care during childbirth among some mothers was also mentioned. It is noteworthy, that in this study, we did not have the perspectives of patients on how they value medical care during childbirth. However, nurses and midwives based on their experience testify that the level of awareness on the benefits of medical care during childbirth is still low in the population, that is why they come to hospital after taking traditional medicines. Nyeko et al. (2016) explored the factors associated with herbal medicine use during pregnancy in Ugandan women [69] and reported an association of cultural beliefs with the use of those drugs. This therefore calls for population sensitization programs on the dangers of use of traditional medicines in pregnancy, as well as the benefits of medical care during childbirth.

### Strength and limitations of the study

The limitation of this study was that nurses and midwives were recruited from only two district hospitals. Therefore, the current study will not generalize findings. However, we believe our findings contribute to an increased understanding of the complexity of PPH management and NR services provision in Rwanda. The strength of this study was a comprehensive sampling of the nurses and midwives with different level of specialization. Both young and more experienced nurses and midwives were represented in the focus group discussions. The interviews were thoroughly analysed to define the three overarching themes and the subthemes. Recruitment was continued until saturation of the data and until any new information was obtained. One FGD included an additional two members who were the maternity matrons and their contribution increased the strength of findings.

## Conclusion

Nurses and midwives were concerned with the survey results and the records review findings. Among possible explanations of the results, the discussions focused on limited in-service trainings, staff shortage, limited clinical guidelines and the lack of some essential materials. Participants also reflected on their current practices focusing on team work, commitment to professional ethics, insufficient skills, learning opportunities, and evidence-based clinical guidelines. Additionally, the study has revealed numerous contextual factors influencing the delivery of BEmONC services in Rwanda with a particular focus on PPH management and NR. The identified factors included; leadership and management, the shortage of staff and heavy workloads, the limited resources, and the profile of clients in relation to their socio-economic status as well as their socio-cultural beliefs and behaviors. Both the self-reflections and the contextual factors are particularly important to recognize and to address when possible, as they significantly affect the quality of facility-based obstetric care. Moreover, our study participants emphasized the need for innovative technology approaches such as eLearning, mLearning, and electronic evidence-based clinical guidelines to help them in continuous learning and clinical decision making at the point of care when dealing with birth complications. Further, the study provides evidence on how nurses and midwives are better sources of context-specific information through situation based-analysis of practices. Our study findings could inform policy reviews to address real practice challenges and promote identified facilitators in order to improve maternal and newborn care services. Also, an in-depth understanding of the reality of practices from the perspective of nurses and midwives, who are the frontline healthcare workers in obstetric care, is essential to enable the design of contextually-targeted interventions to improve facility-based childbirth care in LMICs, including Rwanda.

## Supplementary Information

**Additional file 1.**

**Additional file 2.**

## Data Availability

The dataset generated for this study will be made available from the corresponding author on a reasonable request.

## Abbreviations

BEmONC: Basic Emergency Obstetric and Newborn Care
EmOC: Emergency obstetric and newborn care
LMICs: Low- and middle-income countries
PPH: Post-partum hemorrhage
NR: Neonatal resuscitation
WHO: World Health Organization
UNICEF: United Nations International Children’s Emergency Fund
UNFPA: United Nations Population Fund
FGD: Focus group discussion
CHWs: Community health workers
