# Supplementary material for: Context specific realities and experiences of nurses and midwives in basic emergency obstetric and newborn care services in two district hospitals in Rwanda: a qualitative study
Source: BMC Nurs. 2022 Jan 4;21:9. doi: 10.1186/s12912-021-00793-y (PMC8725506; doi:10.1186/s12912-021-00793-y)
Supplement: Supplementary file 1 — Additional file 1. [file 12912_2021_793_MOESM1_ESM.pdf]

Focus Group Discussion Guide _ Baseline Study

A. Focus Group Discussion Preparation Checklist

The following preparations should be completed and materials obtained before each FGD:

- 12 copies of study information sheets
- 12 copies of consent forms
- 12 copies of audio-recording consent forms
- Box of pens
- Digital audio-recording equipment (tested for working condition)
- Backup batteries for audio recorder(s)
- Notebooks for facilitator
- Private room with at least 12 seats and enough space to arrange seats in a (semi)circle
- Food and drinks for participants

Name and Signature of Study Staff: ________________________Date: ______________________

B. Checklist for Facilitator and Note taker

The FGD will only progress once the following are confirmed:

- All study consent forms have been signed and copies given to participants
- All participants have signed audio-recording consent form
- At least 6 participants in the group
- No more than 8 participants in the group

Note: Participants without the appropriate consent forms or not meeting the inclusion criteria will be excluded.
Name and Signature of Study Staff: _________________________Date: _____________________

C. The questions for Focus group discussions

1. What are the factors influencing the management of post-partum hemorrhage in your hospital? Probe for:
    a. Barriers
    b. Enablers

c. Clinical guidelines
d. Resources available
e. Possible peer-support in the work environment

2. What are the factors influencing the neonatal resuscitation in your hospital? Probe for:
   a. Barriers
   b. Enablers
   c. Clinical guidelines
   d. Resources available
   e. Possible peer-support in the work environment

3. Reflect on the key findings from the knowledge and skills surveys and record review results done in two hospitals (Masaka and Nyamata district hospitals) for the purpose of this research in May 2019. The surveys focused on PPH management and neonatal resuscitation (NR). Our findings indicate that the average knowledge score was 47.2% and the average skills score was 59.9%. With regards to the records review of maternal and newborn outcomes: Apgar score and PPH progressions. 62 % of cases of newborns with newborn asphyxia did not have a stable outcome (some transferred to NCU, others dead) despite 10 minutes' neonatal resuscitation done. While 19% of cases with post-partum hemorrhage were transferred to referral hospitals for further management. Probe for:
   a. Whether Survey results reflect their understanding
   b. Whether Record review results reflect their expectations
   c. Possible explanations of the results

End of the discussion, thank you.
